# Mr. Benjamin Travers on Constitutional Irritation. (3rd and Last Article)

**Published:** 1827-01

**Authors:** 


*An Inquiry concerning that Disturbed State of the Vital Functions usually denominated Constitutional Irritation.* By Benjamin Travers, F.R.S. &c. 8vo. 1826. (3d and last article.)

In the two preceding articles on Mr. Travers's work, there has been little else attempted than an exhibition of practical illustrations, or examples of local and constitutional irritation, without entering into the theory or nature of the subject. This was the course pursued by our author, and we were obliged to follow him in his arrangement, which is the reverse of that usually adopted by writers, who generally state their theories first, and their illustrations afterwards. We do not say that the arrangement of Mr. Travers is inferior to the other—on the contrary, the presentation of those facts, on which a theory is based, prepares the mind for the theory itself, and probably predisposes to an assent which might be withheld if the doctrine were abruptly revealed, without the precession of facts and examples.

In the fifth chapter, then, of the work, Mr. Travers commences his investigation of the theory of irritation, by some observations on the "reciprocal relation of the vital functions." Our author, while he exhibits a decent respect for the experimental investigations of modern physiologists, appears to place little confidence in the conclusions to which they have led. Thus he observes that, although the experiments of Dr. Philip go far towards the presumption of identity between the nervous and electric fluids, yet, "the knowledge of an instrument which serves as a conductor of nervous power, makes feeble approximation to a solution of the problem of life and living actions." And, again—although it appears that some nerves are destined to be conductors of sensation, and others for motion, yet, "to what a monstrous solecism in pathology," says he, "would the doctrine lead, that the *heart is independent of the brain*, because its action continues for a period after the brain and spine are removed—or that the muscular action is a distinct and inherent property of that texture, independent of the nervous, which is only one of its stimuli!" Taking the word *independence* in its common acceptation, as applied to two nations or two individuals, it is perfectly ridiculous, when pressed into the service of physiology, and was a very unhappy expression to select. The heart is no more independent of the brain, than the brain is of the heart. The function of one cannot exist without the other. All the vital viscera, in short, mutually assist and derive support from each other, and the destruction of one must inevitably prove the destruction of the others. But, notwithstanding this mutual dependence on one another, there is still a kind of independence attached to each. The heart has its function to perform, and has its peculiar nerves—so has the stomach, and so on. In perfect health and tranquillity, therefore, these organs appear to carry on their duty with little *interference* from others. But let the function of one be *disturbed*, and almost instantly the functions of all the others are *influenced* in one way or other. Let the heart cease, as in syncope, and see what will become of the sensorial functions—let the cerebral functions cease, and see how long respiration, digestion, and circulation will go on. While we agree with Mr. Travers that "facts of every day's occurrence speak a language far more explicit than that which is extorted by artificial and unavoidably imperfect contrivances;" and that "no result has yet appeared to invalidate the fundamental truth that the brain in totality holds in dependence, immediate or *intermediate*, all the phenomena of life," we are still of opinion that the ganglionic system of nerves holds as absolute dominion over the viscera to which they go, as the brain does over the voluntary muscles. There is, it is true, an intimate connexion established between the two systems, by means of the great sympathetic, the par vagum, the phrenic, &c. so that the one system has the constant means of powerfully influencing the other, without, however, destroying that comparative or qualified independence of which we have spoken. It seems, indeed, a wise provision of Nature that the vital viscera and their functions should have *a degree* of independence of the brain, and *vice versâ*. When morbific agents, however, are applied to either system—sensorial or ganglionic—we find this degree of independence not a whit too great. In short, we would not, perhaps, be far wrong, if we were to assert that one half of the morbid phenomena, presented by the sensorial system, results from the influence of the ganglionic viscera—and that one half of the disorders of these last organs is produced by mental, or in other words, sensorial agency. While physiologists, therefore, go on disputing about the *independence* of these or those organs and functions, the physician can see nothing of such independence; but, on the contrary, the perpetual sympathetic operation of one organ and function on another, throughout the whole chain. This, indeed, is the conclusion to which Mr. Travers, and every practical man comes at last.

"There has been much fallacy, as it appears to me, in Bichat's and other estimates of the comparative influences of the vital functions upon each other; but admitting their general accuracy, to what inference do they lead? Not to that of the independence of either, but the contrary, viz. the inseparable connexion and dependence of each upon the other. In fine, the alliance between the nervous and vascular systems is such as renders it impossible for either to be affected in any serious degree, exclusively." 429.

If the nervous energy be essential to the muscular action of the heart, so is the perpetual current of blood to the functions of the brain. It is, therefore, idle to argue about primordial priority in these two functions. "It may be that *one* is specifically constituted to receive and transmit impressions, both from within and from without, thus establishing the relation of all parts of the system with each other, and of the whole with the material world; and this being unequivocally the case with the nervous system, it must be regarded as that which presides over the vital functions, and through the medium of which they are prompted, regulated, and harmonized." The following appears to be a kind of definition of irritability and irritation, to which we see no objection.

"The irritability of a part conveys to my mind the idea of a sensitive impression and a consequent action. Such impression and action may be strictly local, i. e. not reported to the common sensorium, and under ordinary circumstances, this is the case as regards the involuntary muscular system. The exaltation of this impression under extraordinary circumstances, and its propagation to the brain constituting a sensation, occasions an interference, greater or less, with the habitual actions of parts not subject to the will. Thus extraordinary sensations disturb the habitual action of the heart, liver, bowels, and other viscera. I can see no inconsistency in this explanation of the habitual excitement of the involuntary actions by local and unconscious impressions, with the theory of the universal instrumentality of the nervous system, and it accords with the separableness, to a certain extent and no more, of an involuntary organ, as the heart, from the system at large, without arrest of its ordinary functions.* There is sufficient reason to believe that the system of involuntary nerves is so far independent of the brain, that the ordinary sensitive impressions and actions of the viscera are direct, and not transmitted or performed through its medium. So much may be inferred from their temporary existence in acephali, their correctness in the states of infancy and sleep, and coma, and from the analogy of certain direct habitual actions of voluntary muscles after their separation from the nervous centre, produced by mechanical excitement.† The infinite degrees, varieties, and fluctuations of natural sensation, obvious to our experience, give additional support to the theory of an instinctive or proper sensibility of those parts to their proper stimuli, which are endowed with a power of acting independent of the will." 427.

In the second section, entitled, "derangements of the nervous system, physical and functional," there are scattered a variety of just remarks and accurate observations, shewing the author to be an acute clinical observer—but so unconnected are they, and so void of any clue or thread of arrangement, that we find it impossible to convey, in our own language, a summary of our author's ideas; we must, therefore, have recourse to extracts more frequently than is our custom upon ordinary occasions.

All organs are subject to be affected by remote morbid actions through the medium of the brain—and the brain itself, as the source and centre of sympathy, is especially liable to be affected in its function, both by external and internal causes. Numerous instances of the former class have been adduced by Mr. T. in the preceding articles, and the endless modifications of hysteria present sufficient examples of the latter.

"Frequent instances of sudden death, consequent upon injuries which leave no trace of their destructive operation upon the texture of the vital organs; other instances of death after the lapse of a few hours or days, and some even of weeks from the injury, admit of no other explanation according in any degree with the history and symptoms of the malady, than a suspension or failure of the nervous power. Between those which are of short duration and certain physical injuries of the brain, there is a considerable though not a strict analogy: whereas those which are protracted are characterised by symptoms of exhaustion more nearly resembling such as attend upon changes of structure, in organs auxiliary to the support and preservation of life." 432.

The universal diffusion of the nervous system and its uni-

"* Especially in sudden and violent death, in which case alone the protraction of the heart's motions has been satisfactorily ascertained."

"† Mayo's Comment. Part. II. p. 19."

versal sympathy must be borne in mind in considering these cases.

"It being admitted that injuries and operations are occasionally fatal, which have no relation of contiguity to the vital organs, by inducing certain trains of symptoms which obviously belong to the nervous system, it remains to be inquired, on what principle and in what manner they act destructively. The first and most palpable evidence of impression is that upon the heart. Every thing denotes diminished energy of circulation. In addition to a small and very feeble pulse, we have languor, if not stupor, paleness, chilliness, &c. The nervous influence is capable of acting directly upon the heart either as a stimulus or a sedative. There is no other medium by which an impression, external or internal, can be transmitted. The vital organs are most deeply affected by impressions made over a large surface. Under the permanent depression, as under the privation of the whole nervous function, the properties of sensation and volition, of irritability, and of involuntary motion, cease in succession. The blood, however prepared, cannot excite the heart to action, nor the atmospheric air the organ of respiration, if that irritability or appetency, if I may so express it, which renders them respectively susceptible of and obedient to these stimuli, be exhausted or destroyed." 436.

The *direct* injury of an organ affects its function secondarily—that produced by *sympathy* acts first upon the function. Thus paralysis of the bladder succeeds to a violent blow upon the loins or the abdomen—but it also frequently succeeds to the removal of a tumour, or a spreading inflammation upon any part of the body. In the same way it sometimes happens that the muscles of respiration or the heart itself are similarly affected. Thus a patient has sometimes died upon the table, prior to the commencement of an operation—or immediately after its conclusion. "This is a case of syncope from paralysis or spasm of the heart." Our author knows of no other way of accounting for death in angina pectoris, where no change can be detected in the heart *post mortem*.

On the effects of physical concussion of the brain and nervous system, Mr. Travers makes several just observations. The following extract is worthy of notice.

"It may seem paradoxical to say, that by an injury which has locked up the functions of an organ, the system is preserved from worse consequences; but it is unquestionable that a complete suspension of the sensitive and voluntary powers, when unconnected with læsion of the brain, is a more favourable condition than the state of prostration from shock, indicating a depression of nervous power, so extreme as not to admit of the restitution of healthy actions to sound organs, or inducing that train of deranged actions, and that preternatural mobility of the nervous system which makes it its own prey.*

We are unacquainted, Mr. T. observes, with the essential nature of the injury termed concussion. Some are of opinion that it is never, when assuming a permanent form, uncombined with cerebral læsion, interstitial or superficial, or effusion. "That, to a certain extent, it is complicated with *compression*, is evident from the symptoms common to these injuries, even when most distinct, and the congestive and serous apoplexy." Physical concussion, however, whether cerebral or spinal, (the result of mechanical force directly applied) if not at once fatal, offers, upon the whole, a fairer chance of re-action and recovery than the state of extreme prostration following an injury, in which no violence whatever has been done to the brain.

While of the nature of the nervous agent we confess our total ignorance, we entertain no doubt of the fatality of apoplectic coma, or of phrenzy, although we see both states unattended by any other evidence than that which is present in concussion and in shock, viz. a plethora of the cerebral vessels.

"We are also satisfied to assign as a cause of the most violent convulsions, the irritation produced by an indigestible mass in the stomach, or the resistance which nature opposes to herself in the operation of cutting a tooth. And the same may be said of all the morbid phenomena of the nervous system, and they are many, about which we are perfectly agreed as to cause and effect; although resting solely for the accuracy of the conclusion upon our reiterated experience of their association, and unable to explain upon any known or admitted principle the manner in which such causes operate and such effects are produced. But if it be admitted that an impression of a certain kind upon the stomach, or upon the gum, will produce fatal convulsions without a vestige of physical injury to the structure of the brain, there is surely no difficulty in also admitting that such states as I have described to follow distant injuries, as well as the phenomena of paralysis, spasm, &c. are symptomatic, in numberless instances, of suspended or deranged nervous action, equally independent of any permanent change

"* The difference in respect of the instant effects of injuries happening to persons in a state of unconsciousness from inebriation, sleep, and other causes, is well known, and furnishes an indirect illustration of my meaning. Of two aëronauts who lately fell, owing to the sudden collapse of their balloon, from an elevation of more than a mile in the atmosphere; one was taken up quite dead, the other in a state of syncope, from which she gradually recovered, unconscious of what had befallen her. Although the dead person had fractured his breast-bone by striking the bough of a tree, he had sustained no mechanical injury sufficient to explain his death. His companion had fainted away from terror at his ejaculations prior to their fall, which was the last circumstance she recollected, and her swoon covering the interval of their descent, in all probability preserved her life."

in the structure of the organ. I admit, that the altered action of the heart and vessels of circulation forms the most prominent feature in these cases; but this altered action, like that of the respiratory organ, is due to a primary change in that of the nervous function, the effect of which is universal, though especially manifested in the heart; that organ which is most easily excited or depressed by the operation, stimulant or sedative, of the nervous influence, and whose actions cease only with life." 444.

In injuries of the head and spine, the symptoms are so characteristic that the nature of the seat of the lesion may be ascertained although the history should be concealed from us. If, says our author, the cervical spine be broken adjoining the cranial portion, or above the origin of the phrenic nerves, death is instantaneous from paralysis of the respiratory muscles—"but though more protracted, it is an event *equally certain whatever part of the spine is broken.*"

"I say this with confidence, because I have satisfied myself that the nature of this injury has been generally misconceived. It is a veritable transverse fracture or disruption of the medulla, presenting, on laying open the sheath, a distinct solution of continuity, the fissure or interspace exhibiting, in the first instance, broken down medullary substance mingled with coagula of blood; and if the injury be less recent, a process resembling ulceration. The theca discovers no sign of mischief in a flexure sufficient to break or partially dislocate the vertebræ, and rupture the medulla. Compression, which, from its analogy to the case of cerebral compression, surgeons have concluded to be the cause, seldom exists. Before I ascertained the fact to be as I have above stated, I was surprised to find these symptoms terminating fatally in cases of such partial injury to the bony canal as to present neither a vestige of stricture, nor sign of alteration in the position, direction and appearance of the cord. The mischief is inflicted at the moment in which the injury is received, and is neither perpetuated nor aggravated by any encroachment on the bony canal; consequently the proposal to relieve it by the trephine proceeds, if this statement be of general application, upon an erroneous conception of its nature." 447.

The above passage contains an assertion which we think is much too positive. There are instances on record, and well-authenticated ones too, where fracture of the spine has not been productive of death. If the reader will turn to page 12 of the first volume of this series, he will find the particulars of a case stated by Mr. Harrold, where the spine was fractured—where the patient survived—and where, on dying of another disease, dissection proved the truth of the statement. We need not stop to search for other documents; but surely the above is sufficient to induce Mr. Travers to qualify his assertion. The condition of the spinal marrow, as above described by Mr. Travers, is pointed out in this Journal, Vol. 1, (present series,) p. 10. We are ready to grant that fracture of the spine must be, in 19 cases out of 20, accompanied by irreparable lesion of the spinal marrow; but while a single instance is on record of recovery, after such an accident, we should not entertain the gloomy doctrine which Mr. Travers has here delivered.

When the spinal marrow is actually lacerated or disorganized by the spinal fracture, it was unnecessary for Mr. Travers to account for the fatal issue. The parts below the injury being deprived of their nervous influence soon lose their vital powers, and become incapable of performing their proper functions. The consequences are obvious. With this sketch of the 5th Chapter on the *Theory* of Irritation (if it really deserve the name; for of any thing like a theory we see few traces) we come to the last chapter of the work—entitled "the Pathology and Treatment of Direct Constitutional Irritation."

The first section is on "the State of Prostration without Reaction."

There is reason to believe that a regular series of impressions and actions, maintained between the nervous and muscular systems, is indispensible to animal existence—and that an interruption, derangement, or suspension of these is occasionally the result of a sudden and violent shock, mental or corporeal, or of the two combined, which is fatal or recoverable, according to the greater or less intensity of the shock, and the permanency of the condition from which it originated. This Mr. Travers has called "*prostration*," the term being descriptive, without involving any hypothesis. It is a state nearly approaching a cessation of vital action. Whether this be the direct result of the shock, or of an exhaustive effort of nature to meet the emergency, our author will not take upon himself to decide. It is of more importance to know what is best to be done to rally the flagging power and restore the natural harmonies of the system. The state of the circulation is our chief guide.

"If the shock be unattended by hæmorrhage, and, as the argument supposes, by lesion of any vital organ, immediate or accessary, cordial drinks, as spirituous and vinous liquors and compounds, and what are called diffusible stimuli, as ammonia, æther, camphor, &c. with the aromatic distilled waters, and warm and gently stimulating enemas are the most obvious internal remedies. Externally, the stimulus of artificial heat applied to the pit of the stomach and extremities, as by fomentations; and camphorated and spirituous embrocations, or gentle frictions where the state of parts permits, are the usual means resorted to of exciting the sentient extremities of nerves, and inviting the capillary circulation.

"If any advantage be gained by these means, it becomes necessary to substitute some light aliment for the dram, as gruel, yolk of egg, arrow-root or sago, or to join them, as in jelly, or either of the above substances, with wine or brandy." 456.

We have to bear in mind that it is our object to maintain, but not to force action. The first requires great vigilance on the part of the surgeon. The nurse is incapable of guiding this process—"it is the devotion of a few hours to the life of a fellow-creature."

"Purgative medicine ought not, in my judgment, to be given until the circulation is restored and pretty steady—then a spoonful of castor oil may suffice. It is idly premature to talk about the secretions. Medicines directed to this object cannot act beneficially in such a state of the nervous system, if, as I much doubt, they act at all. It is besides of importance to avoid putting any thing into the stomach but what is essential to support the faultering action of the heart and diaphragm. Since it is upon the stomach we place our chief reliance, we should endeavour by every means to keep it in temper." 457.

We must watch narrowly the patient. If we neglect to supply stimulus the spark of life goes out. As the natural resources come to our aid, we must gradually diminish stimulus and increase nutriment.

The respondence of the circulating forces to an increased supply of stimulus must serve as a caution against over-supply. When the signs of re-action are manifested, its excess is much to be apprehended, if obtained by over-stimulation. Excessive re-action so obtained is "prostration with excitement," in its most perilous form.

"The degree of cerebral affection, independent of any direct injury of the brain, varies so much as to influence materially the symptoms of prostration. The mental faculties are more clouded and stupefied, the respiration is more difficult, and the temperature of the surface sinks in proportion as the cerebral influence is diminished. The muscles are subject to spasmodic and convulsive actions, and the pulse to irregularity and intermissions, in proportion as the cerebrum is, or becomes affected. The 'frissonement' which ordinarily accompanies shock is distinct from rigor.* It is a cerebral affection like the shudder produced by certain acutely painful impressions of the mind or organs of sense. It is not always present, and sometimes wanting where the temperature, to which

"* The distinct origin of tremor from mental and physical impressions is familiarly known. 'You tremble, Sir,' said a by-stander tauntingly to one of the unfortunate victims of the Revolution, on his way to the guillotine. 'True, friend,' he replied, 'but it is from cold, not fear.'"

it seems to have no reference, is to our sensations reduced. 'On the other hand, I have often seen it without any sensible coldness of surface." 559.

It is only at the moment of its occurrence that the direct injury of the brain is liable to assume the common characters of prostration. The stupor, convulsions, and delirium, which characterise injuries of the head by their appearance after an interval in which re-action has been fully established, assume a pathological character very different from those identical symptoms occurring at the moment of injury.

"But if after an injury unattended by hemorrhage, in which the head has suffered no direct mischief, the surface retain its natural warmth, and the pulse and the breathing be oppressed, and there be present stupor, convulsion, and inaction of the pupil; blood should be taken in such quantity as to relieve congestion, and its effect upon the symptoms duly noticed. If, on the other hand, stupor and convulsion be accompanied by a contracted and cold surface, and a thready or intermitting pulse,—I have repeatedly seen both states—the cordial is the proper remedy. Greater freedom of circulation, and a consequent diminution of the stupor and convulsion will be found to follow both one and the other practice, as it is judiciously resorted to." 461.

The custom of blood-letting indiscriminately after accidents is so irrational, and has now been so often reprobated by practical writers, that we would hope it is seldom performed. Our author has seen, and so have we, the arm tied up after a fall or a contusion, when the face was pale, the pulse fluttering, and the breathing irregular. Mr. Travers has known operators permit a large artery to bleed before tying it, in amputations unexpectedly performed on very robust subjects. "It is easy to see the motive of such operators, but it is an erroneous pathology and a dangerous practice; the effects attendant upon loss of blood are peculiar, and the reduction of strength by these means has no analogy whatever to that which is brought about by chronic disease, and renders the effects of operations less formidable."

This, we believe, is sound doctrine. Excepting in some constitutions or conditions, where there is a tendency to hæmorrhage or local congestion in some important organ, blood-letting must be a useless, if not an injurious operation, *there being no excitement present.* This last is the great criterion for venesection, and its effects are far more beneficial than where there is *congestion,* without any evident excitement. In these cases of prostration, opium has appeared inadmissible to our author.

No species of injury exhibits the symptoms of prostration in a more marked degree than extensive burns and scalds.

"The remedy upon which I rely in burns inducing this state, is brandy in gruel, in the proportion of one part of the former to two of the latter. This is administered in doses of a table-spoon-ful or two at short intervals, until we obtain a sufficient distinctness and fulness of the pulse at the wrist, with a corresponding improvement of complexion and temperature of the surface. The bowels should be simply kept unloaded by means of castor oil or common injections. If there be much stupor, and the bowels have been previously costive, I prefer to give ten or fifteen grains of the compound calomel and scammony powder. The use of opium, a remedy still generally employed on account of the patient's sufferings, I have abandoned, being convinced by experience, that in small doses it is inefficacious, and in large ones injurious. I have before observed, that pain is a good symptom in burns. I need scarcely observe that the exhibition of the stimulus requires unceasing and judicious attention; it must be intermitted, if the pulse become bounding, and the patient flush. Nevertheless in deep and very extensive burns of adult subjects, I have frequently found it necessary to persevere in its use for a week or ten days at lengthening intervals; occasionally intermitting, and always gradually withdrawing it. Not only has the partial remission of this plan been in some instances imperative, when reaction was permanently established, but symptoms of the head or chest have made their appearance which required bloodletting, either topical or general; and a smart purge of calomel and scammony I have found on such occasions very useful. In deep burns the external remedy which I prefer is the turpentine and olive-oil liniment, or turpentine and cetaceous ointment, upon the principle, though not exactly the plan, recommended by Dr. Kentish, of Newcastle, and subject, after suppuration commences, to such changes as the state of the surface may require. This appears to me to coincide advantageously with the internal use of the stimulant. The lime-water and milk liniment, and when suppuration has become abundant, the calamine or chalk powder lightly strewed over the whole surface, and defended by the same or simple ointment thinly spread on linen, is the local treatment which I adopt in superficial burns." 465.

Upon the whole, the results of the stimulant plan of treatment in burns, have been highly satisfactory in our author's hands. After comparing the states of syncope and prostration with each other, and proving a strong analogy between them, Mr. Travers proceeds to an analysis of the individual symptoms.

*Nausea.* The stomach is affected with nausea and loathing of food by any sudden depression of nervous energy. This we see in sound health, upon the receipt of sudden moral afflictions, &c. The effect is too instantaneous to be attributed to a deficiency of secretion. Mr. Travers believes it is a direct loss of tone—he would say an erethism of the sentient extremities, and consequently exhalent capillaries of the mucous surface, such as we see a sick stomach and other causes produce upon those of the cutaneous surface. The want, or disappointment of food, palls, and often destroys the appetite. Taking food frequently creates it. Suppressed or depraved sensation is one of the most immediate and remarkable effects of irritation. "It is probable that a similar morbid sympathy to that which suddenly unfits the mind for thought, or the body for exertion, is the cause of that disgust in the stomach which constitutes nausea." This is not only a common consequence, but a never-failing cause of nervous depression.

"While the state of nausea continues urgent, no effective re-action can take place. Vomiting is its relief, and is to the oppressed stomach what rigor is to the oppressed heart. The system, after one full vomiting, experiences a blithesomeness as after the removal of an intolerable burthen; but if the unnatural action be renewed, and become habitual, it is as much a sign as a cause of exhaustion." 469.

*Vomiting*. In muscular organs, Mr. Travers observes, morbid sympathy shews itself in extraordinary action. Thus we explain palpitation, hiccup, vomiting, dysphagia, dysuria, cramp, and spasm.

"The change which a great and sudden depression of nervous energy works in the quantity and quality of the secretions, has undoubtedly a share in provoking the act of vomiting. It would appear that a regurgitation of bile, and its overflow upon the stomach, takes place in these cases as a consequence of the first debilitated, and afterwards deranged or inverted muscular action of the stomach and intestines. Vomiting may be concomitant with a constipated or a lax state of bowels. If it should be attended with constipation, stimulant drastic enemas should be exhibited. If with a free state approaching to looseness, a full opiate enema should be given; and in most cases five or ten grains of calomel with one or more of opium prove advantageous in this state, if retained. A spoonful only of fluid, as brandy and water, or strong tea, or coffee, should be taken by the mouth, and that seldom. A bladder of hot water, a mustard cataplasm, or a blister, should be applied to the pit of the stomach. The more immediate operation of the two former renders them preferable in urgent cases. I have known the calomel and opium pill, and the injection, both cathartic and opiate, selected according to circumstances, stop urgent vomiting, where effervescent saline draughts, acids, alkalies, antispasmodics, cordials, and, in short, every liquid had been rejected from the stomach." 471.

*Hiccup*. This, which is a more advanced and decided symptom of prostration, is a spasmodic affection of the diaphragm, probably from contiguous sympathy with the stomach. We see it generally, but not invariably connected with that irritability which gives rise to vomiting, and both are symptoms almost always with flatulence in the small intestines. Intestinal obstruction, whether directly induced by shock, or indirectly by gangrene, enfeebles, almost to paralysis, the proper muscular motion of the intestines, and thus gives rise to excessive flatulent distention. Hiccup, when of an aggravated character, our author has not known to be arrested—scarcely mitigated. "Its continuance seems to depend, like that of vomiting, on the absolute exhaustion of the nervous energy, the presence of which is as necessary to repose as to action—to control as to excite."

*Rigor.* This is the most uniform announcement of re-action. It is an attendant upon local irritation under a great variety of forms, and is, indeed, the first sign, in general, of febrile excitement. It is a sympathy of the circulating with the sensorial organ—of the heart with the brain. It is produced, however, by various causes, as cold, moisture, fear, &c.; it is a symptom, therefore, of more or less importance, in exact proportion to the cause which has produced it. Arising from a local inflammatory action, as that of suppuration, it is more important than from a temporary exposure to cold or moisture, which does not go the length of inducing fever. Arising from a direct nervous impression, as syncope, or the prostration which is characteristic of severe irritation, it is most important, as the harbinger of returning animation and action, provided it does not, by its violence and duration, destroy re-action, or render it excessive and exhaustive.

### *Prostration, with Excitement and excessive Re-action.*

Re-action may be gradual and restorative of a tranquil state of the system, as after an ordinary fainting fit—or it may be irregular and violent—in which case the subsequent exhaustion may terminate life. This partial re-action is distinguished by its short-livedness, and the non-attainment of a permanent condition, from that in which (the re-action being complete) the more determined and durable character of the symptoms which supervene, implies that it is the result of a positive and continued irritation, either in itself or in its effects.

#### "*Treatment of Prostration with Excitement.*

"The name which I have given to this state sufficiently conveys my opinion that it is based in debility. The energies of the system are diminished in proportion as the actions are increased in cases of excessive re-action. It is a question worthy of our most serious and undivided attention, whether we are in possession of any means of moderating excessive, that is, exhaustive re-action, and of tranquillizing the nervous system, at a time when the greatest disparity exists between power and action. The heart is rather thrilling than fairly pulsating; its semi-contractions are innumerably rapid; the expression is by turns excited and oppressed, wild and comatose; the breathing short, and wanting the relief of sighs. A continued exertion of reason cannot be maintained, and after a correct reply or remark the patient wanders into irrationality. The confusion arising from the rapid alternation of symptoms usually denoting contrary states of the system, and the urgent conviction that death must speedily ensue if this tempest be not assuaged, perplex the judgment of the practitioner. After repeatedly witnessing the total inadequacy of remedies employed under the sanction, and in deference to the axioms of schools and school authorities, I must be permitted to remark that in such a crisis, experimental measures supported by any fair hypothesis should be encouraged rather than met by special objections. If reaction become excessive, and the case assume the character of high excitement, the shaved scalp should be frequently bathed with a cold spirit lotion, a large blister should be applied to the nape of the neck, and the bowels should be cleared with pills of calomel and jalap, or scammony, or, if medicine be rejected from the stomach, by stimulant and purgative injections. The propriety of following up this practice, should it be productive of temporary benefit, is a question of the last importance, for such a state is extremely precarious, and often shifts suddenly into that of sinking.

"For the most part, medicine taken into the stomach, if it be not rejected appears to me to be inert. This organ is not in a state to act upon its contents. Pills are thrown up unaltered an hour or more after they have been swallowed. I prefer in such a case to leave the stomach to itself, and give medicine in the way of lavement. The main question is —should opium be administered in this state? I have seen the highest degree of symptomatic delirium yield to an injection containing three drachms of tincture of opium, repeated after four hours; and by a solution of three grains of the extract in a quarter of a pint of gruel, so exhibited and repeated in the state of incoherent wandering, the mind rendered permanently clear and calm, with a corresponding improvement of other symptoms. In whatever way administered, it is only in full doses that this remedy is admissible in the high excitement of irritation; for larger will be required in such a state to affect, much less to control the nervous system. If it succeed in doing this, its employment is attended with the happiest effects. In the low muttering delirium which accompanies sinking, the frequent repetition of a small dose of opium, as a pill, containing a quarter of a grain every second hour, is decidedly beneficial, as I have seen in many instances.

"The virtues of other sedatives are in my observation pretty correctly appreciated. There is, however, one deserving of particular mention, both as an adjunct to opium, and the best substitute when this is inadmissible. I mean the extract of henbane.* As an anti-irritant it should be given in doses of three to five grains every third or second hour.'' 479.

*Constitutional Irritation from Injuries and Operations.*

After quoting several passages from John Hunter, Abernethy, and Sir Astley Cooper, all of whom, especially Mr. Abernethy, appear to view the irritative fever supervening on wounds and accidents as the necessary consequence of the local disease— "so that, unless this (the local disease) can be altered, we can have no hope of amending the constitutional symptoms," Mr. Travers delivers his own opinion, which is, that the nervous affections are to be regarded as a distinct order of morbid actions (apart from fever or constitutional inflammation) occasionally ensuing upon injuries, upon inflammation, upon the exhaustion attending loss of blood, and upon the admission of noxious matters into the blood. He does not mean to affirm that such actions originate exclusively from these sources, but he selects these as affording prominent examples. Neither does he aver that they may not be combined with febrile action —but if this be the case, they so far pervert or suppress the symptoms of fever as to present a very striking exception to Mr. Abernethy's observation, that "sympathetic fevers are indistinguishable from those not depending on injury."

"It will not, I think, be denied by experienced surgeons, that there is a large, and very important class of cases, in which neither the assemblage of symptoms nor the periodical exacerbations and remissions constituting fever, are present, and which, if we treat them in deference to the rules laid down for the treatment of fever, utterly disappoint and mock our efforts. Of these some never arrive at inflammation, others, owing to the shock which the constitution has sustained, sink almost as soon as inflammation is established, from incapacity to maintain it. Some, as Mr. Hunter has observed, go on well, and when we are no longer in apprehension for the part, the constitution, exhausted by the reparative effort, can go no further, and suddenly collapses; whilst others, displaying from the commencement symptoms of high but irregular excitement, betray in every change, local and constitutional, an excessive and destructive re-action. The first stage of constitutional irritation ensuing upon injury or inflammation, by no means exhibits that provident faculty of nature which is so much insisted upon ; for the tumult of the system, proceeding from the sympathy of the whole with a

"* In the ruffled states of the system generally, but especially in the overactive state of the vascular system, there is a charm in the operation of henbane, altogether peculiar. It is feeble as an anodyne, feebler as a soporific, but not 'poppy nor mandragora' soothe and still so unexceptionably as henbane."

part, is a state with which restorative action is wholly incompatible. The first stage of local irritation or inflammation illustrates this fact. The actions which ensue aggravate the mischief rather than tend to its relief, insomuch that we have often more concern about what is to follow than about what is past, and would gladly compromise for a result. Nevertheless it is not inconsistent to regard the processes, of which these form essential preliminaries, as serving a salutary purpose. But assuredly the first actions, both of the constitution and the part, are those of angry disturbance; and our first business is to compose and allay them. Having fortunately succeeded in this, the actions which require for their institution and maintenance an unusual but not unhealthy vigour, commence as a natural consequence." 487.

Our talented author justly observes that the removal of a mutilated limb, provided the interval of time be so short as to identify the operation with the injury, anticipates, or, at least, infinitely diminishes constitutional irritation. He has, it is true, instanced some cases in which fatal irritation has ensued upon amputation for recent injury—but such cases are rare; while it would be easy to adduce numerous instances leading to an opposite conclusion. "The lapse of a considerable interval in which the system either continues prostrate, or, having in part recovered, acquires a morbid irritability, is exceedingly prejudicial." The recommendation, from respectable authorities, of delaying an operation, on the ground that a certain inurance to confinement and suffering, and the establishment of a suppurative process, so as to gradually conduct the constitution to a state resembling that of chronic disease, will not now be attended to, after the experience gained in the late war. The continued irritation, in fact, of the injury infinitely augments the danger, and the state so much desired and so confidently promised never arrives, in the majority of cases.

"The treatment of deficient re-action and of that which is excessive, resulting from injury or operation, prior to the commencement of inflammation, should adapt itself to the symptoms present in the two states, with this general qualification, that both are evidences of deficient power, and that existing circumstances are often sufficient to create the diversity in the order of symptoms. Thus, hemorrhage determines the one or the other according to its extent. We find the one commonly passing into the other state; and we discover, where in the absence of hemorrhage the former prevails, that the person has been habitually addicted to abuses of constitution; and where the latter, that some strong point of aggravation of the injury, or some extraordinary mental impression explains its predominance. This, it cannot be denied, has often a marked influence—a maniacal tendency for example, or even a high degree of irritability." 490.

In respect to operations for chronic disease, the presence or absence of visceral disorder—the previous habits of life, &c. are points for consideration, and must guide us in the propriety of proposing or dissuading from a surgical operation. The *medical* treatment, also, both before and after an operation, is of much more consequence than the operation itself—though it is a point too little attended to, either by the student or the operating surgeon. We recommend the following passage to the student who neglects his medical studies for the purpose of running from hospital to hospital, to get a glimpse of operations which he may never be called upon to perform—or, if performed, which he will not know how to treat afterwards.

"The practice of performing a serious operation, and leaving the after-treatment to others, has, in my knowledge, repeatedly proved disastrous. The medical treatment, a duty not less responsible than the operative, belongs to the surgeon, and indeed, to be employed merely as a handicraftsman, conveys an imputation at which the dignity of a scientific mind revolts." 492.

If students were to attend strictly to the patients in hospitals, after operations, they would learn much more useful knowledge than they do—but the knife is so captivating, that they will run from one end of the town to the other to see it brandished, and seldom afterwards take the pains to see the result. Yet they will never learn to operate by the sight of operations. A good knowledge of anatomy, and the sight of a few operations, will enable them to do much more than a slight anatomical knowledge, with the sight of a multitude of operations.

### *Constitutional Irritation from Inflammation.*

Inflammation is strong and effective, or the contrary, as it is free from irritation or mixed with it. Sympathetic fever may be of the pure inflammatory character, bold and powerful, or it may be of vacillating and weakly character. It may be sympathetic synochus, or sympathetic typhus. The *latter*, says Mr. T. though it differs in character from the former, is still a mode of sympathetic fever, and hence is treated, primarily at least, by medicines addressed particularly to the secreting organs.

"The irritation set up by inflammation has generally more or less of febrile action blended with it. But irritation, in my notion of the term, is not fever under any modification. Fever requires, and as Mr. Hunter justly observes, shews powers of resistance. In irritation we have no determinate or continued train of actions. It is 'every thing by turns and nothing long.' Teachers appear to me to confound irritation with inflammation or fever, and because they sometimes co-exist, to conclude they coincide. Irritation may be a symptom of fever, as fever may be an effect of irritation, but they are originally and essentially distinct forms of disease, and either may exist in the absence of the other.*

"Sympathetic fever is a healthy re-action of the constitution, without any impairment of its powers, such as they are, shewn in the increased energy of the vascular system. If the natural powers of the constitution be deficient, the fever assumes the low or typhoid character, and in its progress gives out indications of the disturbance and distress of the nervous system from its inability to support the increased action of the vascular; but the nervous affections are secondary, strictly symptomatic of the fever, whatever be its origin, and therefore indistinguishable from those which attended upon low fever in the absence of injury." 494.

In the treatment of inflammatory fever ensuing upon complicated injury, the chief indication, Mr. Travers observes, is to tranquillize action without materially diminishing power. "We ought not to be alarmed at fever, but at the want of it." We ought not to use any rough and permanently depressing means to put it down, neither blood-letting nor continued profuse purging and sweating.

"To moderate extraordinary action it is sufficient to keep up such a determination to the secreting organs, as prevents their becoming so obstructed as to increase fever. I am persuaded that practitioners are generally over anxious to cut short sympathetic febrile action, and treat fever from sudden and artificial, too much as they treat fever from gradual and natural causes. The great bearing of the constitution, under a sudden call upon its powers, is decidedly upon the nervous system. To a certain extent the existence of fever, for which an obvious and adequate cause appears, is less trying to that system than the employment of such measures as are necessary to its sudden abridgement. For if the inflammatory action be marked and vigorous, the nervous, which supports it, is strong and inirritable, and the former cannot be suddenly pulled down without a serious encroachment upon the strength and steadiness of the latter." 496.

By these observations Mr. Travers does not mean to adopt the Hippocratic practice, lately so general on the continent, of leaving every thing to Nature, and of passively watching

* "I am aware that in this remark I have to contend with the difficulty to which all writers on pathology and on many other subjects are exposed, arising from the vague and various meaning affixed to terms in common use, according to their vulgar acceptation. Thus if any reader should be startled at the idea of fever devoid of irritation, I trust to his discrimination and candour to do justice to my distinction between irritation as an archetype or original disease, and irritation as a symptom."

her operations whether salutary or the contrary. He only means to say that, the less violent our measures, the better. Nervous power is much more easily depressed than raised. Irritation is a very possible result of treatment as well as of malady—for by those means which lower the system too rapidly, we incur the danger of converting inflammation into irritation, and thus of destroying our only medium of recovery.

Some management is necessary in respect to the local irritation. The part injured should be kept in an easy position, with light and soothing applications. The swelling and tension, which are the signs of local re-action, should not be aggravated and rendered a source of additional irritation, by too nice adjustments and contrivances. The application of lint, moist or dry, to a fresh wound, quickly becomes an irritant; and adhesive plasters, circular bandages, and splints, applied as they too often are, operate as strictures or ligatures.

"Plugging a fresh wound to force suppuration, or strapping it to prevent that process, are equally liable to be followed by injurious consequences. The attempt to render complicated wounds simple by leaving them in the original close dressings for many days in succession, has proved a common, and sometimes fatal mischief. I have seen more than one case in which the constitutional irritation arising suddenly, and proving fatal in a week from the injury, was distinctly referrible to a diffused erysipelas and gangrene, set up by such local irritation and stricture. A sufficient inspection of the part at intervals of two days, to satisfy an experienced eye of its well doing, may be accomplished, not only without detriment to the process of healing, but with manifest relief to the patient, and is a widely different thing from handling and disturbing the position of parts, against which I would be among the first to protest as uncalled for and injurious. Penetrating wounds attended with hemorrhage, and comminuted fractures with extensive injury of the soft parts, and in the vicinity of large joints, are especially the cases which present themselves to my mind in offering this remark." 498.

The change which parts undergo in passing from a state of health to a state of gangrene, is not greater, our author observes, or more remarkable, than that of the nervous system from a healthy condition to that of extreme prostration. Ulceration and acute gangrene sometimes exhibit the state of excessive re-action, terminating in exhaustion. Mr. Travers attended a young West Indian lady who had a blister applied to the knee-joint for a strumous inflammation. It was purposely irritated to suppuration, with the intention of being kept open; but the constitution being feeble, an ulcerative process supervened, and rapidly destroyed the integuments down even to the ligaments. Stimulants and nutriment failed to save the young lady's life. The following practical inference terminates this section of the work.

"The general inference which I would draw, relating to the treatment of sympathetic irritation, as compared with that of sympathetic fever, is as follows, viz. the symptoms of excitement belonging to the first, although resembling those of the second, are in point of fact allied to a powerless state of constitution, and will not bear to be treated after the same manner. Blood-letting, general and topical, and a forced increase of the secretions by active forms of medicine frequently repeated—the appropriate remedies for high inflammatory fever—are calculated to aggravate, in an alarming degree, the symptoms of irritation. In partial exception to this general observation, however, it sometimes happens that the effect of suspended biliary secretions is evidenced in an obstinate confinement of the bowels, and in this case the relief of the symptoms is not obtained until copious bilious evacuations have been procured. The aloe or colocynth extract, or blue pill combined with calomel, and repeated as required, are the best medicines with which I am acquainted for relieving this state. Alcohol, ammonia, æther, camphor; opium and henbane; and bark in substance, decoction, and tincture, are the articles upon which, in the circumstances respectively indicating their use, I believe most reliance may be placed in the treatment of acute sympathetic irritation." 501.

Mr. Travers had not, we suppose, made trials of the sulphate of quinine, in such cases, when this part of the work was composed. Its salutary effects, we are confident, will be found far superior to those of any of the medicines above enumerated.

### *Constitutional Irritation from Loss of Blood.*

Mr. T. is afraid that prostration, pure or mixed, the result of hæmorrhage, has not been an uncommon product of the intemperate use (or rather abuse) of the lancet, the practitioner's judgment having been led astray by the temporary relief obtained even to the last by the detraction of blood in acute diseases. Some persons cannot bear the loss of blood—it gives rise to prostration, attended with convulsions, in which the circulation fails so alarmingly as to require watching for several hours, and the repeated administration of stimulants to restore it. Here our author passes a high, and a well merited eulogium on the essay published by Dr. Marshall Hall, "On the Effects of Loss of Blood," "which, (says Mr. Travers) for the general accuracy of its descriptions, and the reasoning by which they are illustrated, is, in my view, highly creditable to the author's talents as a pathologist."* We have given such

* We are happy to find that this talented physician has taken up his an ample account of Dr. Hall's essay, in a former number, that we cannot revert to it in this place.

The effect of hæmorrhage upon the nervous system is two-fold—immediate and remote.

"The *immediate* is syncope, and is in proportion to the suddenness rather than the quantity of the hemorrhage; hence the relative effect of the same loss in quantity is greater and more frequently fatal from the large trunks and near the heart, than from the small vessels and at a distance from it. The *remote* effect is to precipitate and contract the actions of the heart, and is in proportion to the quantity lost, as in frequently recurring hemorrhages or a slow protracted hemorrhage; and this effect as well as the former, depends upon such an atony, from deficient excitement, of the nervous system, as incapacitates it for maintaining the actions of the vascular." 506.

If a single hæmorrhage, Mr. T. observes, has been so great as to deprive the system of the material whence nervous excitement and nutrition are derived, "the transfusion of a portion of human blood seems a fair and rational expedient," since it is clear, from Dr. Blundell's experiments, and subsequent trials, that a much smaller quantity supplied than that which has been lost, will suffice for this purpose. "But if the system be, at the same time, so depressed by the shock, or previously so exhausted by hæmorrhages or by any chronic disease, that the loss of a moderate quantity of blood induces the state of pure prostration, it is extremely doubtful if a fresh supply would renovate its powers, or be attended by any beneficial effect." In this sentiment we entirely agree.

"When prostration with deficient re-action is the result of hemorrhage, support by nutriment in the most assimilable forms, given in moderate quantities at short intervals, and by nutritive clysters, is the most important indication. To obtain the equal influence of the attenuated circulation and to tranquillize the nervous system, a pure and fresh atmosphere, the preservation of a natural and warm surface, the sedulous exclusion of every cause of excitement, and a very cautious use of vinous stimuli, the danger of serous effusion being much augmented in this case, are the only means left us to pursue." 507.

The fifth and last section in this excellent work is on "constitutional irritation from the absorption of animal poison," and the greater part of the section is occupied with observations on the cases of Dr. Bell, of Plymouth dock-yard, and Dr. A.

---

residence in the metropolis, where his abilities will have a more ample scope than in a provincial town. We prognosticate that this zealous and gifted physician will experience that reception among his brethren here, which his merits deserve. Public favour is sometimes lavished on those who are little entitled to it. It will not, in this instance, however, be ill bestowed.

Thomson of Sloane Street. As we have given such detailed accounts of these cases in preceding numbers of this Journal, tis unnecessary to dwell upon them here. The narrative of Dr. Bell's case confirms our author in the belief that he was the subject of a distinct and specific irritation, viz. that of a poison, of which, allowing for mental and other influences, he would, at any other time, have been a sufferer—perhaps a victim.

"If this opinion be correct, it follows that the local action proper to this state is an unessential feature of the disease with which we have to contend; the disease itself consisting in a direct prostration of the vital forces, marked by an excitement of the most transitory description, and rapidly terminating in collapse or exhaustion." 514.

We may refer to our review of Dr. Butter's work, vol. 4, p. 70, for nearly similar sentiments. In respect to Dr. Thomson's case, published in the Medical Repository for April, 1825, Mr. Travers considers it a clear case of thecal puncture and inflammation going on to suppuration, "in which the ordinary signs of constitutional irritation were modified by the existing state of health, which was considerably below the ordinary standard." Again, our author observes, "a comparison with the genuine cases of absorption will demonstrate that *this* was not one of that class; and a reference to some of the cases which I have related, of fascial and thecal inflammation will sufficiently account for the impression made on the nervous system." This distinction, we apprehend, will not be admitted by all persons, nor by the patient himself, Dr. Thomson, who evidently attributed the phenomena to a virus received into the system during dissection.* When we consider, indeed, that, in the course of 12 hours after a scratch, so minute as to pass unnoticed at the time it was made, there arose a train of *constitutional* symptoms of the most formidable description, it requires no small stretch of the imagination to attribute all this to local inflammation of theca or fascia, without any reference to a poison received. Mr. Travers, in fact, acknowledges that, "the symptoms of extreme constitutional irritation arising from simple and specific causes are not in their nature distinguishable, without the aid of *certain associations* of local characters." In the first place we conceive that few, indeed, would be able to satisfy themselves respecting a distinction so extremely difficult to make;—and, in the next place, if they *were* able to discriminate by means of "certain associations," and where the "symptoms of extreme constitutional irritation"

* Vide Vol. 3 of this series, p. 253-4-5-6.

were so much alike as to be indistinguishable from each other, would the treatment differ in the two cases? We apprehend that the extreme physical and intellectual prostration presented in Dr. Thomson's case, must have required the same cordial and stimulant treatment, whether the cause were thecal inflammation or a specific virus. When the diagnosis turns on so nice and difficult a point as that represented by our author, we believe that symptomatology is our surest guide, leaving etiology, for a time, out of the question—especially when great uncertainty prevails respecting the nature of the morbid cause.

Mr. Travers introduces the details of a case in this place, which is of recent occurrence, and "of much practical value, as it illustrates more forcibly than any, the supervention of the specific upon the simple inflammation, and a variety of the former, probably thence resulting." The case is rather minutely detailed, and we must abridge it materially.

*Case.* A young gentleman, of delicate and irritable habit, grazed the point of the fore-finger of his right hand, with the blade of his scalpel, while opening the body of a man, three hours after death from extravasation of urine. He immersed the part in water, and proceeded with the examination. In returning home he shivered, but regarded the circumstance as occasioned by the cold air, (January 1826.) His digestive organs had been, for some time, out of order—and his mind was anxiously bent on his studies. Next day (8th January) he felt unwell—kept the finger immersed in cold water—and took a calomel pill going to bed. He passed a restless night, and next morning the absorbents of the arm were inflamed up near to the axilla. The fore-finger was swelled, tense, and painful on pressure. An incision down to the bone was made at the tip of the finger, which bled freely. He was now looking and feeling very poorly, with a hurried pulse, depression of strength, and anxiety of mind. The evacuations were found to be unhealthy, and therefore three grains of calomel and a grain of opium were given at bed-time, followed by a morning aperient. On the 3d day, he was rather better—took some liq. ammon. acet. mist. camph. and spir. æth. nit. in the day, repeating the calomel and opium at night. Hand and arm freely bled by leeches. *4th day.* Considerably more sunk by constant nausea and retching. Some carbonate of ammonia and laudanum added to the medicine prescribed the preceding day. Extract of henbane at bed-time. There was diffuse swelling with inflammation of the arm, the inflammation of the absorbents having disappeared. *5th day.* The erythema had spread, and there was a patch of inflammation on the biceps—the general symptoms the same, the nausea still continuing, with occasional hiccup, and wandering delirium. *6th day.* The inflammation had extended to the neck, and over a small part of the pectoral region. Large vesications had appeared on the back of the elbow and hand. Leeches again applied to the inflamed surfaces. Three grains of carbonate of ammonia and one of opium hourly—camphor draught to be continued—anodyne fomentations. *7th day.* More composed—pulse 120, soft and powerless—tongue getting brownish—hiccup—erythema faded on the hand and arm, vivid on the chest.

We need not farther detail this case; the symptoms gradually increased, and this unfortunate youth sunk on the eleventh day from the receipt of the injury.

Another gentleman in good health assisted at the same dissection. He punctured his finger—the wound inflamed—but yielded to a poultice and some opening medicine.

Our author has adduced this case for the purpose of shewing an additional instance of the combination of the simple and specific action, or rather, how the latter may succeed to the former, and suspend it after an interval of two or three days.

"The possible and not very unfrequent co-existence of the signs of simple inflammation, or in the commencement of these cases, their separate and exclusive appearance, renders the diagnosis embarrassing, and the additional circumstances of their having a common origin from dissection, and the constitutional symptoms being, in some instances indistinguishable, greatly add to the embarrassment. At one time doubts existed as to the real nature of the case just detailed. They gave way however to the weight of evidence in favor of its specific origin. *But the existence of a doubt confirms the important fact, that the state of constitution indicates a mode of treatment in all respects the same.*" 527.

The latter part of this passage, which we have taken the liberty of marking in italics, confirms the sentiments which we expressed a page or two back, respecting the treatment. The following passage is still more explicit on this important point.

"A poison admitted by a wound or raw surface, and a poison admitted by the lungs, are equally excitants of a specific constitutional irritation; which shows itself in the latter case in a specific mode of inflammation, if the individual should unhappily become the subject of the slightest injury. The distinction between the erythema of poison, and that mode of inflammation following a wound, appears to me to be this: that the first is distinct from the mechanical condition, the second grafted upon it. The first is the action of the poison viâ constitution, showing itself in one or more points upon the line of parts by which it has entered the system; and whether it take place in a visible shape or not, depends upon the intensity of the poison, the powers of the constitution, the duration of life, &c. The second is the conversion of the healthy into an unhealthy inflammation, depending either upon a peculiar local irritation, or upon the peculiar temperament or circumstances of the individual. Hence the analogy is so close between the cases of constitutional origin, direct and indirect, as in some rare instances to render the distinction, so far as the constitution alone is concerned, inappreciable; but upon the pathology and treatment this analogy reflects a valuable light, inasmuch as it warrants the inference that the resemblance is essential, and that a similar rationale, and by consequence a similar plan of treatment are to be adopted. Accordingly, the history of these cases and of the effects of different modes of treatment confirms the inferences, that they are accompanied by a remarkable depression of nervous energy, shewing itself either in pure prostration or prostration with excitement, and exhaustion; that all reduction of power is a violation of the leading indication, and that the antiphlogistic measures adapted to simple inflammation and its sympathetic fever are therefore positively contra-indicated; that the action of the arterial system requires to be sustained in order to its being equalized; that pain and every incidental excitement is, if possible, to be assuaged, and repose, by whatever means, to be procured." 529.

A considerable number of these unfortunate cases have now been made public—and it is probable that a still larger number are kept in the back ground, for reasons more selfish than scientific. It is remarkable, Mr. Travers observes, that, of those presenting the *specific* character, the instances of recovery have been very rare—perhaps not more than one in seven; while, of those "blended and confounded with them of absorbent and glandular inflammation, including the cases of cellular and fascial inflammation, and of suppuration in veins and thecæ, the fatality is certainly not one in twenty." We coincide with our distinguished author in the following conclusion to which he has come.

"That the effect of the introduction of a virus, whether secreted by serous membranes under inflammation, or formed in all animal bodies during the first stage of decomposition, is local inflammation, and the production of a similar virus in the inflamed part, as Dr. Thomson thinks, appears to me a conclusion unwarranted by the majority of the cases which I select as examples of the specific disease. For in few instances can the part wounded be said to undergo inflammation, at least sufficient for secretion; and the characteristic inflammation usually takes place at a greater distance of space, and at a shorter interval of time, than would, according to analogy and probability, happen, if the virus were a morbid poison elaborated in the wound. The case of a vesicle or pustule formed on the wound, is probably an exception to this remark." 531.

In respect to local diagnosis, Mr. T. observes that, in no case which has fallen under his notice, has the sign of pain more or less acute, and of fulness and tenderness on pressure of the breast or shoulder, been wanting. The erythema may not shew itself till the fourth or fifth day; but it more frequently develops itself on the third. It appears on the pectoral, scapular, or iliac region, running towards the median line, and bounded by it.

"It is not symptomatic of deep-seated inflammation, nor does the cellular membrane take on the full suppurative action, until the disease has attained considerable duration, and then diffusedly. I regard this, including the stages of pain, puffiness, and efflorescence, with or without the vesicle, as the surest local and visible character of the disease. Its locality is in the first instance determined, by the passage of the irritant into the circulation, corresponding to that of the previous pain. As it becomes more completely a constitutional action, after the lapse of some days, it is subject to become erratic, and appear not only on the flank or loin on the same, but even on the opposite side of the trunk, or opposite limb, without any obvious cause of local irritation." 532.

Many plans of local treatment have been proposed for dissection wounds. Dr. Pett's case is a warning against the use of caustic after the commencement of inflammation and pain. It is clear that if an escharotic is to be applied at all, it should be at the moment of the injury, considering the rapidity of absorption in these cases, as compared with that in hydrophobia. The following is a sensible observation.

"Since it cannot be known, at the moment of infliction, what may be the nature of the injury, it is as little to be expected that young men will habitually apply a caustic, on the instant, as that they will smear their fingers with oil or pomatum, or clothe them with gloves before handling a body. Neither are such things, in my opinion, worthy of men in earnest in their pursuit; but I must take leave to deprecate the absurd and affected hardihood of persevering in dissection, and especially in visceral examinations, with recently cut or chapped fingers affording every facility for absorption; and thus needlessly exposing themselves to the imminent risk of a disease from which so few, even of the most robust, escape with life." 536.

When inflammation is once set up, the applications should be simple and soothing. If much tension be present, the vessels should be emptied freely by a deep incision through the line of the wound, and then the part enveloped in a poultice. For the erythema of the arm or trunk, folds of linen moistened with the diluted liq. plumbi, or liquor ammoniæ acetatis, are probably the best applications. If, in protracted cases, the cellular membrane becomes much loaded, incisions are beneficial, to quicken the slow and imperfect suppurative action. It appears to our author, as indeed to every judicious and observant practitioner, that no rules for the constitutional treatment, as applicable to all cases, can be laid down—in fact, that we should equally err in uniformly prescribing a stimulant or a depressing treatment.

"As regards the first, I feel warranted in saying that the plan of early support formerly adopted in these cases of animal poison, under the notion, however erroneous, of a rapid putrescency of the fluids, is more consistent and rational than the antiphlogistic, which assimilates them to inflammation. Venesection, it is true, is one mode of relieving congestion; but a more pernicious one could not be devised, where the congestion is the obvious result of a sudden and extreme depression of nervous power." 537.

The above sentiment is almost precisely what we have always professed, when treating of this subject in various parts of this Journal. Mr. Travers remarks that, should a case present itself bearing the specific local character, in which acute pain was combined with steady contracted pulse, and other signs of inflammatory excitement, he "would consider the trial of copious blood-letting in the commencement indicated, nay, called for;" but he never met with such a case. Where the use of the lancet is prohibited, pain should, if possible, be allayed by opium. Our author wisely advises the practitioner to be guided, not by preconceived opinions, but by existing indications. When he speaks of existing indications, he does not mean to refer to this or that symptom, but to the assemblage of symptoms. With the following extract we shall conclude our extended analysis of this work.

"The impression arising from a belief that a solitary symptom is sufficient to constitute a guide, is a most mistaken one: for example, a rapid pulse, if frequency be considered an insuperable objection to tonics; a wandering or asthenic delirium, if, as is generally thought, it forbids the use of opium; a white-furred tongue, which I have heard intelligent physicians declare prohibitory to the exhibition of bark, &c. As to frequency of the pulse, it is oftener a sign of weakness than the opposite; and I know no period of acute irritation at which it would be possible to institute a tonic regimen, if this amount to a prohibition. In local inflammations, the benefit of astringent and tonic remedies is lost, if their use be deferred until the elevated sensibility of increased action has entirely subsided, and the action is becoming habitual to the part or chronic. And so it is with the constitutional excitement of irritation, which begins in weakness. It is the active and sensitive stage alone, upon which any favourable impression is to be made by medicine. I offer this observation to enforce the distinction between action as a sign of power, and action as a proof of weakness. Again, for the state of mild delirium, or rather the state of prostration, of which it is characteristic, my experience teaches me that opium, in certain proportions and combinations, oftener than any other medicine affords a remedy. If a peculiar state of the tongue, whatever that may be, counter-indicates the early employment of bark and ammonia, it sets aside the remedies of which I entertain a higher opinion than of any, in the prostration of irritation from external causes.*" 541.

---

"* As I have mentioned bark—in a firm belief of the virtues of which medicine I agree with Mr. Hunter, a belief founded on some experience—it is right to say that my mind is yet undecided on the actual and comparative

It is of very little consequence, either to the public or the author, what may be the opinion of a reviewer respecting a work, provided the analytical delineation be copious, and the author's sentiments impartially and fully stated. Whenever the critic means to injure an author and deceive the reader, he goes upon a diametrically opposite plan. He is either very sparing of quotations, and liberal of his own opinions—or he selects only the weak points for exhibition, and draws a veil over every thing of a redeeming quality. It is all "carbone notamus," with such reviewers; and there is, unfortunately, a strong principle in human nature, which appears to receive pleasure both in the infliction and contemplation of injustice—hence the most flagrant and illiberal criticisms are those which excite most *amusement* in the minds of the readers. But amusement and instruction are two different things. The former affords a momentary gratification, and is not again referred to—indeed, by its very nature it will not bear repetition. But with instruction it is otherwise. It will not only bear retrospection, but improve in value on reflection. It is under this impression that our analytical labours are conducted, believing that by setting forth a full, fair, and impartial analysis of a work, we act the part of a good judge, who exercises *his judgment* most, in laying before the jury such a faithful representation of the case as enables *them* to pass the final judgment between the two parties. That we have enabled our readers (a great majority of whom can never see the original) to form a just estimate of Mr. Travers's work, and, consequently of his opinions and practice, we hope will be acknowledged—and this is the desideratum on all sides. As we have, on several occasions, and on particular points of doctrine or practice, expressed our individual opinions freely, whether as corroborating or opposing the sentiments of the author, so we cannot see the necessity, or even the propriety, of giving any thing like a summary decision at the conclusion. This final sentence, indeed, is the proper attribute of a higher tribunal—the PUBLIC; and its assumption is a dangerous prerogative, more frequently claimed with arrogance, than exercised with talent and integrity by the critic.

---

merits, in the more urgent cases of this class, of the preparation lately imported from France, under the denomination of sulphate of quinine. It is the misfortune of every new remedy to be put upon the forlorn hope, and thus to encounter a very unfair mode of trial. Its efficacy however in the cure of ague appears to be satisfactorily established."

